# Assessment of Cardiorespiratory and Metabolic Contributions in an Extreme Intensity CrossFit^®^ Benchmark Workout

**DOI:** 10.3390/s24020513

**Published:** 2024-01-14

**Authors:** Manoel Rios, Klaus Magno Becker, Filipa Cardoso, David B. Pyne, Victor Machado Reis, Daniel Moreira-Gonçalves, Ricardo J. Fernandes

**Affiliations:** 1 Center of Research, Education Innovation and Intervention in Sport and Porto Biomechanics Laboratory, Faculty of Sport, University of Porto, 4200-450 Porto, Portugal; up201803272@up.pt (K.M.B.); up201402398@edu.fade.up.pt (F.C.); ricfer@fade.up.pt (R.J.F.); 2 Research Institute for Sport & Exercise, University of Canberra, Canberra 2617, Australia; david.pyne@canberra.edu.au; 3 Department of Sport Sciences, Exercise and Health, University of Trás-os-Montes e Alto Douro, 5001-801 Vila Real, Portugal; victormachadoreis@gmail.com; 4 Research Center in Sports Sciences, Health Sciences and Human Development, 5001-801 Vila Real, Portugal; 5 Research Center in Physical Activity, Health and Leisure, Faculty of Sport, University of Porto, 4200-450 Porto, Portugal; danielmgon@fade.up.pt; 6 Laboratory for Integrative and Translational Research in Population Health, 4050-091 Porto, Portugal

**Keywords:** oxygen uptake, bioenergetics, total energy expenditure, CrossFitters

## Abstract

Our purpose was to characterize the oxygen uptake kinetics (VO_2_), energy systems contributions and total energy expenditure during a CrossFit^®^ benchmark workout performed in the extreme intensity domain. Fourteen highly trained male CrossFitters, aged 28.3 ± 5.4 years, with height 177.8 ± 9.4 cm, body mass 87.9 ± 10.5 kg and 5.6 ± 1.8 years of training experience, performed the Isabel workout at maximal exertion. Cardiorespiratory variables were measured at baseline, during exercise and the recovery period, with blood lactate and glucose concentrations, including the ratings of perceived exertion, measured pre- and post-workout. The Isabel workout was 117 ± 10 s in duration and the VO_2_ peak was 47.2 ± 4.7 mL·kg^−1^·min^−1^, the primary component amplitude was 42.0 ± 6.0 mL·kg^−1^·min^−1^, the time delay was 4.3 ± 2.2 s and the time constant was 14.2 ± 6.0 s. The accumulated VO_2_ (0.6 ± 0.1 vs. 4.8 ± 1.0 L·min^−1^) value post-workout increased substantially when compared to baseline. Oxidative phosphorylation (40%), glycolytic (45%) and phosphagen (15%) pathways contributed to the 245 ± 25 kJ total energy expenditure. Despite the short ~2 min duration of the Isabel workout, the oxygen-dependent and oxygen-independent metabolism energy contributions to the total metabolic energy release were similar. The CrossFit^®^ Isabel requires maximal effort and the pattern of physiological demands identifies this as a highly intensive and effective workout for developing fitness and conditioning for sports.

## 1. Introduction

Quantifying the dynamic features of oxygen uptake (VO_2_) kinetics has gained popularity in human physiology as a means of identifying the mechanisms underlying the control of muscle VO_2_ during exercise [1,2]. Traditionally, the dynamic VO_2_ response to exercise has been studied in three intensity ranges, i.e., low-moderate (until the anaerobic threshold) [3], heavy (above the anaerobic threshold) [4] and severe (in the area in which maximal VO_2_ is achieved) domains [5]. More recently, the extreme exercise domain has been proposed for performances leading to exhaustion before maximal VO_2_ is reached, with VO_2_ kinetics characterized by the development of a fast component with insufficient time for the appearance of a discernible VO_2_ slow component [6]. VO_2_ kinetics at low-severe exercise intensities have been well established in cyclic exercise modes including running, cycling, swimming and rowing [2,7,8,9].

**Figure 1:**
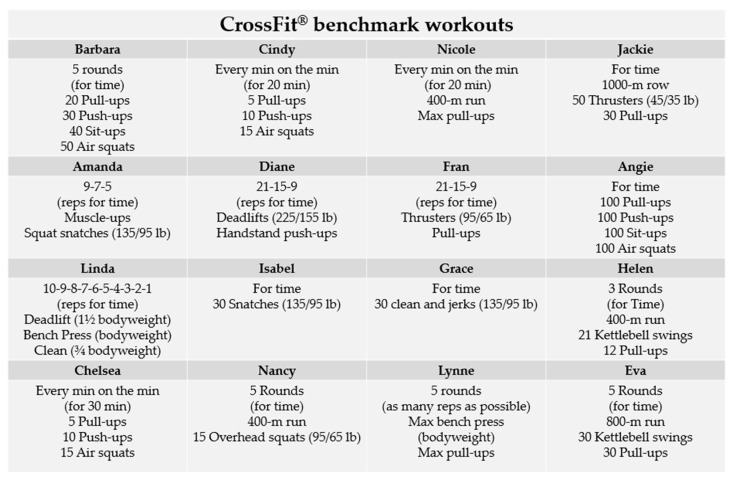
Overview of exercises and structural elements in CrossFit^®^ benchmark workouts.

CrossFit^®^ is a multi-modal physical training program that covers functional movement patterns in a single high-intensity training session emphasizing strength and metabolic conditioning [10]. Improvements in metabolic capacity and lung function provided by CrossFit^®^ are functions of the duration, type and intensity of exercise [11,12,13]. To assess and monitor fitness while tracking changes in work capacity over time, CrossFit^®^ has integrated standardized exercises known as benchmark workouts [14,15]. These benchmarks exhibit variations in specific exercise routines/composition, intensity, duration, number/type of exercises and rest periods (see Figure 1) [16]. The manipulation of these parameters will ultimately affect the magnitude of the fitness and performance improvements, and the associated risk of overload [11,12]. Scoring methods typically involve achieving a total number of repetitions within a specified time frame [16]. CrossFit^®^ research is needed to uncover predictive variables for performance in benchmark workouts through conventional laboratory tests [17,18,19]. However, due to the specificity of these workouts, the application of traditional laboratory testing protocols is constrained, as the physiological demands diverge from those encountered in a real-world training context. It is worth noting that only a limited number of pre-established workouts have been systematically characterized, with Cindy [20,21,22] and Fran [11,12,13,17] benchmarks workouts standing out as the most extensively assessed.

Prior studies reveal an acute blood oxidative stress [23] response and heightened concentrations in the indirect blood markers of muscle damage, such as interleukin-6 and creatine kinase, post-CrossFit^®^ sessions [24,25,26]. Diverse CrossFit^®^ workouts correlate positively with elevated blood lactate concentrations ([La^−^]), highlighting the impact of factors like workout intensity, duration, exercise variety and rest periods on physiological responses [11,13,21,22,27]. In CrossFit^®^ workouts conducted at severe intensity, there is a substantial oxidative phosphorylation energy contribution that should be considered a vital element in the training process. In addition, the optimization of various metabolic pathways is achievable depending on the total duration and performance (intermittent or continuous) strategy of the workout [11,13,21]. Furthermore, an observed reduction in muscle functional capacity underscores the dynamic nature of these workouts [21,22]. Cardiorespiratory and bioenergetic assessments have been primarily assessed in well-controlled environments, particularly in exercise laboratories [28,29]. The number of studies conducted on training and competition conditions is limited [11,13]. Studies not accounting for the oxygen-independent metabolism (glycolytic and phosphagen pathways) contribution at higher exercise intensities result in an underestimation of the total energy expenditure, which negatively impacts the overall understanding of the effects of specific workouts [30].

Isabel stands as a timed CrossFit^®^ benchmark workout, challenging participants to complete 30 snatch repetitions with a 61 kg barbell in the shortest time possible. Widely employed for evaluating performance improvements in CrossFitters, this workout is renowned for its substantial muscular power demands [31]. However, to gain a more comprehensive understanding of the effects of workout intensity on the specific training performance of CrossFitters, more detailed physiological assessments, such as [La^−^] and cardiorespiratory parameters, are essential [11,12,13,21]. The purpose of the current study was to characterize the VO_2_ kinetics, estimate the contribution of the different energy systems and calculate the total energy expenditure of the Isabel workout. We expected that the specific cardiorespiratory demands would be consistent with the extreme intensity domain classification.

## 2. Materials and Methods

### 2.1. Participants

Fourteen highly trained male CrossFitters of 28.3 ± 5.4 years old with height 177.8 ± 9.4 cm, body mass 87.9 ± 10.5 kg, lean body mass 52.1 ± 4.5%, fat body mass 12.3 ± 4.8%, body mass index 27.8 ± 2.2 and 5.6 ± 1.8 years of training experience volunteered to participate. Subjects were recruited if they had a CrossFit^®^ training frequency of more than five times per week for a minimum of three years before the commencement of the study. Participants were contacted personally and selected based on the following eligibility criteria: (i) ability to perform the Isabel workout <2 min; (ii) age between 18 and 40 years; and (iii) eligibility to exercise according to the Physical Activity Readiness Questionnaire. All CrossFitters were provided with clear instructions to adhere to their typical nutritional habits and explicitly instructed to abstain from consuming alcohol and caffeine, as well as engaging in intense physical activity, in the 48 h prior to the test. Detailed information about the experimental procedures, associated risks, and the benefits of participation was provided to all volunteers. All experiments were approved by the local Ethics Committee (CEFADE212019), with participants reading and signing an informed consent form in accordance with the Declaration of Helsinki and guidelines of the World Medical Association for research with humans.

### 2.2. Experimental Design

**Figure 2:**
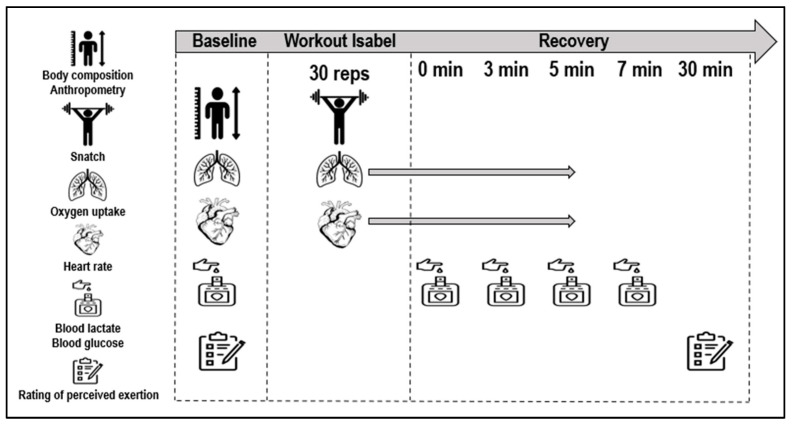
Workout Isabel data collection set-up.

All assessments were conducted in a gym facility, maintaining consistent environmental conditions of 23 °C ambient temperature and 60% humidity. The assessments were supervised by an experienced CrossFit^®^ researcher, ensuring meticulous and precise execution, thereby maintaining consistency and reliability across all participants. Initial measurements of body mass were obtained using the InBody 120 (Seul, Republic of Korea), while height was recorded using the Seca 222 stadiometer (Brussel, Belgium) immediately upon participants’ arrival. A standardized 10 min warm-up, including joint mobility exercises and specific movements with low loads tailored for Isabel, was administered. Subsequently, each CrossFitter engaged in the Isabel workout, exerting maximal effort. Pulmonary gas exchange was monitored breath-by-breath throughout the baseline, during the workout and in post-workout phases using a K5 telemetric portable gas analyzer (Cosmed, Rome, Italy). Simultaneously, continuous heart rate data were captured by a telemetric heart rate monitor belt (Cosmed ANT+), transmitting information to the K5 portable unit (Figure 2). During the recovery, subjects maintained a seated position for subsequent data collection. Capillary blood samples (5 μL) were collected from a fingertip at baseline at the 1st, 3rd, 5th and 7th min post-workout. The initial sample was discarded to eliminate contaminants and ensure measurement accuracy. Capillary blood collection involved applying controlled pressure to the finger, minimizing volume variations for consistent results. Lactate concentration ([La^−^]) and glucose levels were determined using the Lactate Pro analyzer (Arkay, Inc, Kyoto, Japan) and Accu-Chek Aviva analyzer (Mannheim, Germany), respectively. The participants’ self-reported perceived exertion was assessed using the Borg scale ranging from 6 to 20 (from very, very light to very and very heavy) at both baseline and 30 min post-workout. To ensure accurate interpretation and consistent reporting, the rating of the perceived exertion scale was explained individually to the participants according to the recommendations [32].

### 2.3. Methodology

**Figure 3:**
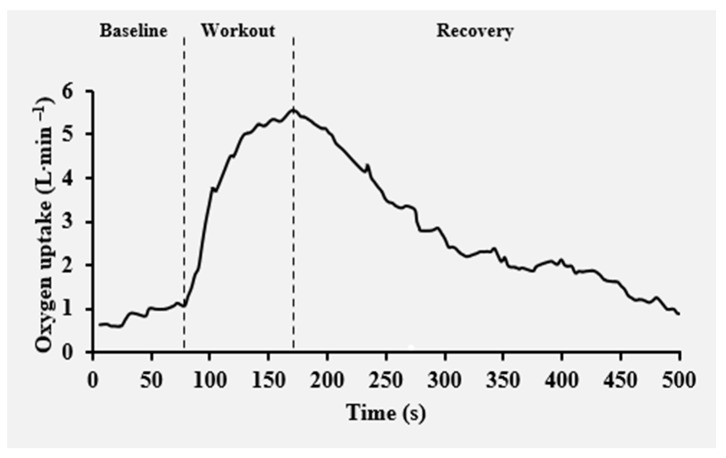
Example of individual oxygen uptake kinetics as a function of time along the baseline, Isabel’s workout and recovery.

The VO_2_ peak and ventilatory variables’ mean values were determined by analyzing the data from the final 30 s of exercise [13]. Data were carefully reviewed, and any breaths resulting from coughing or signal interruptions were excluded from the analysis [33,34]. Only values within the range of mean ± 3 standard deviations were considered for further analysis. Subsequently, a smoothing process was applied using a moving average for three breaths and a temporal average for 10 s, respectively [35]. For the estimation of VO_2_ kinetics parameters, a bootstrapping approach with 1000 samples was employed, with the exclusion of the cardiodynamic phase from the analysis [36]. The on-transient VO_2_ of Isabel’s workout and the excess post-exercise VO_2_ were determined using both mono-exponential and bi-exponential models through the VO_2_FITTING software [37]:

$$VO_{2}(t)=A_{0}+H\;(t\;-\;TD_{p})\;A_{p}\;(1\;-\;e^{-(t-TD_{p})/\tau_{p}})$$

$$VO_{2}(t)\;={\;A}_{0}-H\;(t\;-\;TD_{p})\;A_{p}\;(1\;-\;e^{-(t-TD_{p})/\tau_{p}})\;-\;H\;(t\;-\;TD_{\mathrm{sc}})\;A\mathrm{sc}\;(1\;-\;e^{-(t-TD_{\mathrm{sc}})/\tau_{\mathrm{sc}}})$$

where VO_2_(t) represents the oxygen uptake normalized to body mass at time t, which is the baseline value for VO_2_, H denotes the Heaviside step function, Ap and Asc are the amplitudes of the primary and slow component phases, whereas TDp and Tsc, τp and τsc are the corresponding time delays and time constants of the fast and slow components of VO_2_, respectively [37]. Accumulated VO_2_ was computed as the ratio of the time integral of net VO_2_ to the exercise duration [11]. An individual example of the VO_2_ kinetics during and post-exercise responses is presented in Figure 3.

To assess the contribution of the oxidative phosphorylation energy system, the time integral of the net VO_2_ versus time relationship was examined [11,13]. The oxygen-independent metabolism contribution was approximated as the sum of the energy derived from lactic acid production and phosphocreatine splitting in the contracting muscles [11,38]:

$$\mathrm{Glycolytic}\;\mathrm{pathway}=[La^{-}{]}_{\mathrm{net}}\cdot \beta \cdot M$$

where [La^−^]_net_ is the peak accumulation of lactate after exercise, β is the constant for O_2_ equivalent for lactate accumulation in the blood (3 mL·kg^−1^·mM^−1^) and M (kg) is the body mass of de CrossFitter [11,38]. The phosphagen pathway contribution was estimated based on the maximal phosphocreatine splitting in the contracting muscle. This estimate assumed an energy equivalent of 0.468 kJ·mM^−1^ and a phosphate/oxygen ratio of 6.25 [11,34]:

$$\mathrm{Phosphagen}\;\mathrm{pathway}=\mathrm{PCr}\;\cdot \;(1-e^{-t/\tau })\;\cdot \;M$$

where t represents the time duration, τ is the time constant of phosphocreatine splitting at the onset of workout (23.4 s), M (kg) denotes the mass of the participant and PCr is the assumed phosphocreatine concentration at rest, set at 18.5 mmol·kg^−1^ [34,38]. Energy system contributions were quantified in kilojoules (kJ), assuming an energy equivalent of 20.9 kJ·L^−1^ [11,39]. The total energy expenditure during Isabel’s workout was estimated by summing the contributions of the three energy systems [38,39]. To estimate metabolic power, energy expenditure was divided by the total duration (s) of the Isabel workout [11,38]. Caloric expenditure is estimated by multiplying absolute VO_2_ by 5.05 kcal·L^–1^ (expressed in kJ by assuming an energy equivalent of 4.184 kJ·L^−1^) [11,33].

### 2.4. Statistical Analysis

All calculations were completed using GraphPad Prism 6, with descriptive statistics presented as mean and standard deviation (SD). Data normality was checked through the Shapiro–Wilk test and repeated-measures ANOVA (with a Bonferroni post-hoc test) was applied to compare cardiorespiratory and energetic variables at different time points. A paired sample *t*-test was applied to compare perceived exertion and metabolic variables before and after the workout. Based on a post-hoc analysis, a sample of 14 subjects, an effect size of 0.8 and a 0.05 overall level of significance, the statistical power (β) obtained was 0.80. The effect size was calculated using Cohen’s *d* and interpreted as follows: trivial if *d* < 0.2, medium if 0.2 > *d* < 0.5 and large if *d* ≥ 0.5. The statistical significance level was set at 5%.

## 3. Results

**Figure 4:**
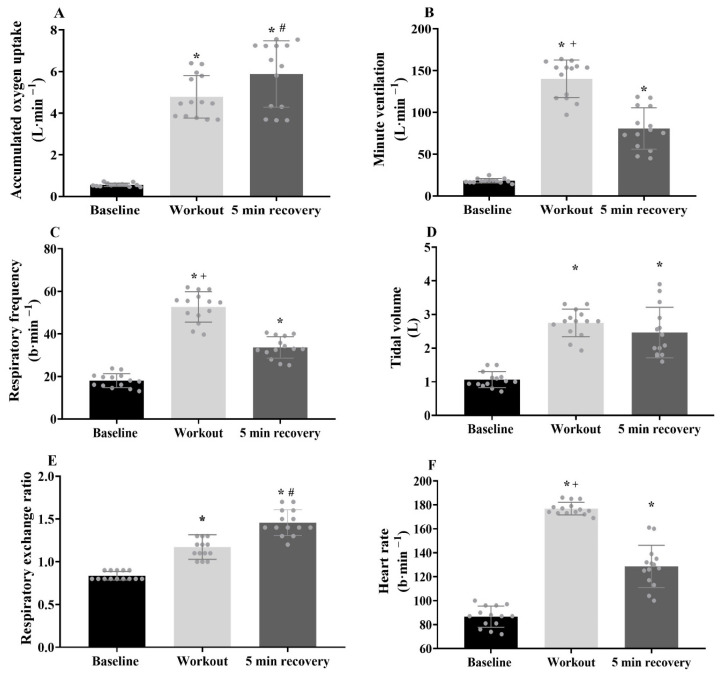
(**A**–**F**) Cardiorespiratory variables assessed during baseline, Isabel workout and recovery with the respective differences identified by *^, #, +^, respectively (*p* ≤ 0.05). Individual and mean ± SD values.

The overall Isabel workout duration was 117 ± 10 s, with an exercise frequency of 0.3 ± 0.0 repetition/s. During the exercise, the VO_2_ peak, primary component amplitude, time delay and time constant values were 47.2 ± 4.7 and 42.0 ± 6.0 mL·kg^−1^·min^−1^, 4.3 ± 2.2 and 14.2 ± 6.0 s (respectively). The cardiorespiratory values at baseline, during the Isabel workout and at recovery are presented in Figure 4. The accumulated VO_2_ (*p* = 0.001, d = 5.8), minute ventilation (*p* = 0.001, *d* = 7.6), respiratory frequency (*p* = 0.001, *d* = 6.2), tidal volume (*p* = 0.001, *d* = 5.1), respiratory exchange ratio (*p* = 0.001, *d* = 3.3) and heart rate (*p* = 0.001, *d* = 12.3) values were substantially elevated from baseline. The excess post-exercise VO_2_ (*p* = 0.001, *d* = 4.7), minute ventilation (*p* = 0.001, *d* = 3.5), respiratory frequency (*p* = 0.001, *d* = 3.6), tidal volume (*p* = 0.001, *d* = 2.5) and heart rate (*p* = 0.001, *d* = 3.0) values remained elevated at the post-workout 5 min of recovery compared to baseline, with excess post-exercise VO_2_ (*p* = 0.026, *d* = 0.8) and respiratory exchange ratio (*p* = 0.001, *d* = 1.8) values greater than the exercise condition. In contrast, minute ventilation (*p* = 0.001, *d* = 2.5), respiratory frequency (*p* = 0.001, *d* = 3.1) and heart rate (*p* = 0.001, *d* = 3.7) were higher along the exercise compared with the recovery period. Regarding metabolic variables, there was a 14-fold increase in [La^−^] and a 46% increase in glucose levels in post-exercise values compared to baseline. The perceived exertion was much higher at the 30 min of recovery than at baseline (Table 1).

**Figure 5:**
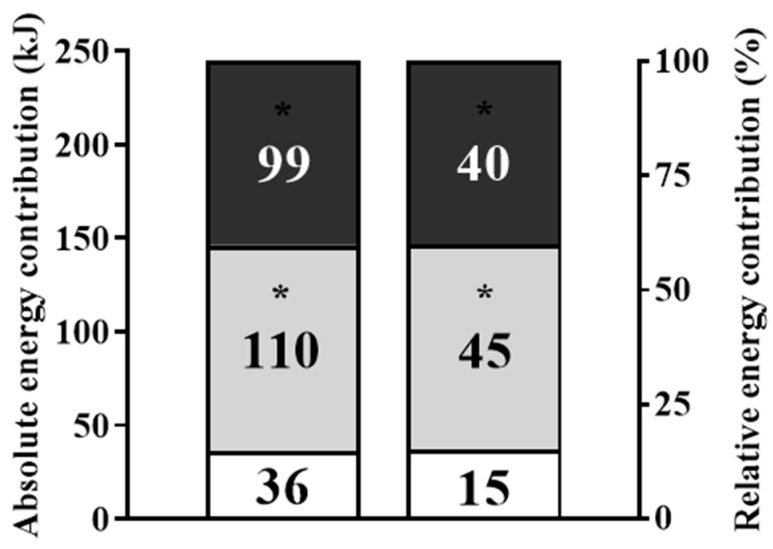
Isabel workout (absolute and relative) oxidative phosphorylation, glycolytic pathway and phosphagen pathway energy contributions (identified by dark grey, light grey and white). * Differences from phosphagen pathway (*p* < 0.001).

The absolute and relative energy contribution values for the total workout are presented in Figure 5, with the oxidative phosphorylation (*p* = 0.001, *d* = 4.0) and glycolytic pathway (*p* = 0.001, *d* = 4.7) systems values contributing substantially more than the phosphagen pathway system (with higher values for glycolytic pathway contribution than the oxidative phosphorylation system). Total energy expenditure and metabolic power values during the Isabel workout were 245 ± 25 kJ and 2.0 ± 0.2 kW, and the caloric expenditure was lower during the workout than during the recovery period (101 ± 22 vs. 124 ± 34 kJ; *p* = 0.026, *d* = 0.8).

## 4. Discussion

We characterized the VO_2_ kinetics, estimated the energy system contributions and evaluated the total energy expenditure of the Isabel CrossFit^®^ workout. This exercise is typically performed in ~120 s and generates energetic demands in the extreme intensity domain [6]. Our main findings are summarized as follows: (i) a fast VO_2_ increase occurred at the beginning of the workout and continued to rise during the exercise yielding a high accumulated VO_2_; (ii) a greater contribution of the oxygen-independent metabolism (~60% of the total energy release) was observed (as a sum of both the glycolytic and phosphagen pathways); and (iii) the total energy expenditure values were high. The velocity of muscle contraction during the workout resulted in higher cardiorespiratory and metabolic stress (evidenced by the excess post-exercise VO_2_, [La^−^] and glucose values) compared to baseline, which ultimately affected the return to homeostasis. These high metabolic demands confirm the utility of the CrossFit^®^ Isabel workout as a very effective high-intensity training modality for enhancing fitness and conditioning.

It is well established that performing an a priori test in a standardized protocol, such as an intermittent incremental treadmill test, allows the collection of [La^−^] analysis and, in conjunction with gas exchange assessments, provides a comprehensive physiological characterization of exercise in the low, moderate, heavy and severe intensity domains [8]. However, advocating an a priori standardized testing protocol to determine maximal VO_2_ (e.g., cycling, treadmill and rowing ergometer) and comparing it to the Isabel performance that includes specific strength training elements compromises the principle of modality specificity in sports training. For this reason, we chose not to perform a priori tests to determine the maximal VO_2_ of CrossFitters. Instead, we compared their values with cardiorespiratory data from athletes participating in sports performed at the same intensity.

Although VO_2_ kinetics is well described in the literature, especially in cyclic sports [2,9], few attempts have been made in the CrossFit^®^ literature to evaluate VO_2_ kinetics using direct oximetry protocols under real exercise conditions. When the Isabel workout is performed at maximal effort, CrossFitters begin exercising at a very high intensity. From the onset of the exercise, the requirement for oxygen in muscles triggers an instantaneous and sudden increase in the accumulated VO_2_, resulting in a high peak VO_2_, which is consistent with recently reported data obtained in trained CrossFitters performing the CrossFit^®^ Fran workout [11,13]. The current primary component amplitude and time constant values were similar (but with smaller values for the time delay) than those previously reported for rowing, running and cycling at maximal intensity [39]. Faster VO_2_ kinetics is related to a shorter time lag in the imbalance of VO_2_ demand and supply, implying an increased oxidative contribution to energy transfer [40]. In addition, given that the CrossFitters were trained, it likely influenced a shorter time constant (associated with higher fatigue tolerance) contributing to better performance [40,41].

Cardiorespiratory outcomes during the Isabel workout yielded similar values for minute ventilation, respiratory frequency, tidal volume, respiratory exchange ratio and heart rate compared with running and cycling at maximal intensity [8,39]. However, the accumulated VO_2_ and heart rate values were lower than those obtained in the previous evaluation of the Fran workout [11,13]. The differences in accumulated VO_2_ values can be explained by the greater exercise volume involved in this latter effort (90 repetitions of thrusters plus pull-ups) that ends up demanding a greater pulmonary function [11,13]. In support of this assertion, the short duration of the Isabel workout (combined with the extreme intensity) limited the increase in heart rate and prevented it from reaching a higher value [12]. The [La^−^] and glucose concentrations in response to the extreme intensity of the Isabel workout were higher than the Grace and Fran CrossFit^®^ workouts [13,20], reflecting a greater glycolytic pathway contribution and involvement of carbohydrate metabolism. These effects would likely reflect a greater amount of glucose at the muscle level [30]. The perceived exertion was lower in the Cindy [21] and Fran [11] workout sessions compared to the Isabel workout, which was classified as extremely hard. A higher rating of perceived exertion value during an intense workout session indicates that sufficient stimuli are present to promote resistance adaptation, as the rating of perceived exertion value has been used as a marker of psychophysiological response to a training session [42].

The oxidative phosphorylation contribution determined in the current study was lower than the values previously reported for Fran workout [13], running, cycling [39] and rowing [43], but higher than the values reported for strength training [35]. In contrast, the oxygen-independent metabolism (phosphagen and glycolytic pathways) contribution during Isabel workout in the present study was higher than that reported for the other types of training [11,43], although these differences could be attributed to the shorter total duration (117 s) and high net [La^−^] accumulation (1.5–20.7 mmol∙L^−1^) of the Isabel workout [30]. In addition, specific mechanical factors (e.g., the muscle contraction scheme and the resulting muscle fiber recruitment profile itself) might have influenced the energy contribution of the workout, which in turn largely depends on the type of training performed [33]. Nevertheless, some caution should be exercised when interpreting data, as different methodological procedures can easily influence the energy contribution [30]. The gold standard for assessing oxygen-independent metabolism release involves a highly invasive muscle biopsy, quantifying energy sources and metabolite accumulation inside muscle cells. However, the technique’s limitation lies in sampling only a small portion of human muscle tissue, requiring multiple samples from different depths to reflect muscle heterogeneity [34].

Studies that have examined total energy expenditure, metabolic power and caloric expenditure assessment under CrossFit^®^ training conditions, particularly at extreme intensity, are few in number. Total energy expenditure during the Isabel workout was lower than the Cindy and Fran workouts [11,20], but higher than those reported for strength training [44] and rowing [43]. Metabolic power reported in this study was higher compared to the Fran workout [11] and rowing [43], while caloric expenditure was lower compared to strength training [45], Cindy [20] and Fran workouts [11,13]. These differences could be due to intensity, volume, the number of repetitions and different types of exercises [33], and provide a framework for the prescription of training in these settings.

Cardiorespiratory function remained elevated during the recovery period (as expected) compared with baseline values, consistent with other exercises [13,46]. This elevation is justifiable because strength training can induce Valsalva maneuvers and an increase in cardiovascular demands, which may yield a compensatory rise in minute ventilation and VO_2_ during the recovery period [47,48]. In addition, the higher minute ventilation and VO_2_ could also be interpreted as part of the compensation to normalize the lowered pH caused by increased [La^−^] levels post-workout [46,48]. The higher respiratory exchange ratio during the recovery compared to baseline and exercise conditions is likely explained by the explosive nature of the Isabel workout. The Isabel requires a rapid production of adenosine triphosphate via the oxygen-independent metabolism (60%), resulting in a greater involvement of carbohydrate metabolism [30].

Our results indicate that Isabel’s workout, completed in less than two min, triggers a substantial and effective cardiorespiratory response. This regimen could serve as an excellent training option for optimizing both oxygen-independent metabolism and oxidative phosphorylation pathways. Certain limitations in our study warrant acknowledgment. First, the relatively small number of participants and the absence of dietary control before the test must be acknowledged. Caution should be used when extrapolating the results of the current study to other cohorts or individuals with different training experiences, as only healthy, experienced, male participants were recruited for this study. Several variables, including active muscle mass percentage, a time constant of VO_2_ on the response at the muscle level and the concentration of phosphocreatine splitting per kilogram of wet muscle, can influence the values obtained using the phosphagen pathway method [34]. Finally, this study lacks biochemical markers (e.g., creatine kinase, total antioxidant status and malondialdehyde) to elucidate the elevated physiological stress induced by a single training session of Isabel.

In future cross-sectional studies, comparing cardiorespiratory responses and energy utilization between novice and experienced CrossFitters is valuable. Furthermore, future research should assess the metabolic profile of novice participants to understand the impact of strategies employed during the Isabel workout in this cohort. It is crucial to explore variations in these responses between males and females, emphasizing adaptations in both central (stroke volume) and peripheral (oxidative capacity) aspects. Longitudinal studies analyzing the effects of prescribed training interventions over weeks in response to the consecutive days of CrossFit^®^ training, using similar methods and including biochemical markers and biomechanics analysis, are crucial for a comprehensive understanding of how to accurately quantify and monitor CrossFit^®^ training load.

## 5. Conclusions

During the extremely intense Isabel workout, there was an immediate and sudden increase in VO_2_ at the beginning of the exercise that persisted until the end, highlighting the contribution of oxidative phosphorylation energy metabolism during short and very intense CrossFit^®^ workouts. The majority (~60%) of the total energy was obtained from oxygen-independent metabolism, and both the glycolytic and phosphagen pathway energy systems should be strengthened to improve the performance of trained male CrossFitters. The Isabel workout is an excellent high-intensity training option for CrossFitters and other athletes seeking to improve their fitness and conditioning.

## Figures and Tables

**Table 1:** Baseline and Isabel workout metabolic demands and perceived exertion.

Variable	Baseline	Isabel	*p*	*d*
Peak blood lactate (mmol∙L^–1^)	1.5 ± 0.3	20.7 ± 2.6	0.001	9.8
Peak blood glucose (mg∙dL^–1^)	97.1 ± 4.6	141.8 ± 8.6	0.001	6.2
Rating of perceived exertion (6–20 scale)	6 ± 1	18 ± 2	0.001	7.5

Mean ± SD, probability (p) and effect size (*d*).

## Data Availability

All data are contained within the article.
